# Visualizing the enteric nervous system using genetically engineered double reporter mice: Comparison with immunofluorescence

**DOI:** 10.1371/journal.pone.0171239

**Published:** 2017-02-03

**Authors:** Yanfen Jiang, Hui Dong, Lars Eckmann, Elaine M. Hanson, Katherine C. Ihn, Ravinder K. Mittal

**Affiliations:** 1 Division of Gastroenterology, Department of Medicine, School of Medicine, University of California, San Diego, California; 2 San Diego VA Health Care Systemm, San Diego, California; Wayne State University, UNITED STATES

## Abstract

**Background and aims:**

The enteric nervous system (ENS) plays a crucial role in the control of gastrointestinal motility, secretion and absorption functions. Immunohistochemistry has been widely used to visualize neurons of the ENS for more than two decades. Genetically engineered mice that report specific proteins can also be used to visualize neurons of the ENS. The goal of our study was to develop a mouse that expresses fluorescent neuronal nitric oxide synthase (nNOS) and choline acetyltransferase (ChAT), the two proteins expressed in 95% of the ENS neurons. We compared ENS neurons visualized in the reporter mouse with the wild type mouse stained using classical immunostaining techniques.

**Methods:**

Mice hemizygous for ChAT-ChR2-YFP BAC transgene with expression of the mhChR2:YFP fusion protein directed by ChAT promoter/enhancer regions on the BAC transgene were purchased commercially. The Cre/LoxP technique of somatic recombination was used to construct mice with nNOS positive neurons. The two mice were crossbred and tissues were harvested and examined using fluorescent microscopy. Immunostaining was performed in the wild type mice, using antibodies to nNOS, ChAT, Hu and PGP 9.5.

**Results:**

Greater than 95% of the ENS neurons were positive for either nNOS or ChAT or both. The nNOS and ChAT neurons and their processes in the ENS were well visualized in all the regions of the GI tract, i.e., esophagus, small intestine and colon. The number of nNOS and ChAT neurons was approximately same in the reporter mouse and immunostaining method in the wild type mouse. The nNOS fluorescence in the reporter mouse was seen in both cytoplasm as well as nucleus but in the immunostained specimens it was seen only in the cytoplasm.

**Conclusion:**

We propose that the genetically engineered double reporter mouse for ChAT and nNOS proteins is a powerful tool to study of the effects of various diseases on the ENS without the need for immunostaining.

## Introduction

The enteric nervous system is important for the control of gastrointestinal motility, secretion, absorption, sensation and immune functions. During embryonic development, the neural crest cells of the central nervous system (CNS) migrate into the gastrointestinal tract to form the enteric nervous system (ENS). The latter is organized into two major plexuses, myenteric/Auerbach and submucosal/Meissner, and several minor plexuses. Each of these plexuses are made up of ganglia (nodes) that are connected to each other with the internodal strands. Each ganglion is a collection of many different types of neurons that can be classified based on the, 1) morphological appearance, 2) electrophysiological properties and, 3) chemical or neurotransmitter content [1–3]. The myenteric plexus resides between the circular and longitudinal muscle layers and is mostly responsible for the control of gastrointestinal motility. The majority of the myenteric plexus neurons can be divided into excitatory and inhibitory, which cause contraction and relaxation of the longitudinal and circular muscle layers. Acetylcholine (Ach) and substance P are the major neurotransmitters of excitatory motor neurons. On the other hand, nitric oxide (NO) and vasoactive intestinal peptide (VIP) are the major inhibitory neurotransmitters of inhibitory motor neurons.

For more than 20 years, investigators have used immunohistochemistry and histochemistry to identify various neurons of the enteric nervous system. The works of Brookes[4–6] in guinea pig, Sang in mice[7] and Wattchow in humans[8–10] show that majority of the myenteric neurons of small and large intestine contain either choline acetyl transferase (ChAT), the enzyme responsible for the synthesis of acetylcholine, or nitric oxide synthase (NOS), the enzyme responsible for the synthesis of nitric oxide. In fact, more than 95% neurons of the myenteric neurons are positive for either ChAT or NOS, a small number (<5%) positive for both, and the remainder for neither NOS nor ChAT. Immunostaining of neurons, while a powerful technique, is cumbersome and does not always provide reproducible results [11]; it can be operator dependent and affected by the quality of antibodies used.

For more than 10 years, genetic approaches to visualize myenteric neurons have been developed. Several transgenic mice that express fluorescent proteins in subset of neurons of the myenteric plexus have been described [12]. Mice with fluorescent cholinergic neurons of the ENS have been described by several investigators and are commercially available. However, to the best of our knowledge there are no report of mice with fluorescent NOS protein, and combined fluorescent ChAT and NOS proteins. The goal of our study was to develop a reporter mouse that expresses nNOS alone, and both ChAT and NOS fluorescent proteins. Such a mouse can be a powerful tool in the study of ENS because one would be able to visualize 95% of the myenteric plexus neurons using fluorescent microscopy without the need for antibody and immunostaining. We compared data from the reporter mouse with immunostaining method in the entire gastrointestinal tract, i.e., esophagus, small intestine and large intestine.

## Material and methods

The Institutional Animal Care and Use Committees at the Veterans Affairs San Diego Healthcare Systems and University of California, San Diego approved the study protocol. All experiments were conducted in accordance with the Guidelines for the Care and Use of Laboratory Animals (National Institutes of Health, Bethesda, MD). Mice were scarificed by dislocation of cervical spine (for freshly tissue samples) or perfusing with 2 to 4% paraformaldehyde under deep anesthesia using a cocktail containing ketamine (3.75 ml, 100mg/ml), xylazine (0.4 ml, 100mg/ml), and acepromazine (0.75 ml, 10mg/ml) mixed with 15.1 ml distilled water. Mice hemizygous for ChAT-ChR2-YFP BAC transgene with expression of the mhChR2:YFP fusion protein directed by the choline acetyl transferase (ChAT) promoter/enhancer regions on the BAC transgene were purchased from the Jackson Lab, (stock#012355) and bred at UCSD. To construct mice with nNOS positive neurons, the Cre/LoxP technique of somatic recombination was used. Mice expressing Cre-ERT2 recombinase under the control of the nNOS promoter (knock-in mutation) were crossbred with Cre reporter mouse positive for red fluorescent protein, Td-Tomato. The Cre-ERT2 fusion gene activity was induced by administration of tamoxifen, so only cells that express Cre (i.e., nNOS positive cells) produced the fluorescent reporter. The two fluorescent reporter strains for ChAT and nNOS were cross bred to generate the double-reporter mice. The two fluorescent reporter proteins have different spectral properties, i.e., emission at 527 nm for YFP and 581 nm for Td-Tomato.

### Tissue processing and fixation

The mice that confirmed positive for both ChAT and nNOS were treated with oral tamoxifen, 1 mg/day for 2 days, after which either fresh tissue was obtained from the animals or they were perfused with 2–4% paraformaldehyde under deep anesthesia. For fresh tissue, the entire gut, from the esophagus (at the level of cricoid cartilage) to the distal rectum was harvested. The mucosa of the harvested segments, i.e., entire esophagus, fundus of the stomach, small intestine (20mm proximal to the ileocecal valve) and colon (20mm distal to the ileocecal valve) was removed and the muscularis propria was mounted on a glass slide with 99% glycerol and examined under the microscope. The perfusion was performed though the left ventricle with 20 ml saline followed by 4% paraformadehyde in 0.1M phosphate buffer, pH 7.4. The entire gut was removed in a manner similar to the fresh tissue and examined under the microscope.

For immunostaining, wild type mice were perfused and tissue harvested as described earlier, post-fixed overnight in 4% paraformaldehyde at 4°C. Samples were then soaked in 30% sucrose solution in 0.1M phosphate buffer overnight. Samples were treated with 0.3% TritonX-100/1%BSA/PBS over night at 4°C to make them permeable as well as block non-specific staining. Samples were placed on a shaker, washed 3 times with PBS, each time for 15 minutes and then primary antibodies (Table 1) were applied for 48 hours at 4°C, following which tissue was washed again 3 times (15 minutes each) before incubating with secondary antibodies (Table 1) for 1 hour at room temperature. Finally the samples were mounted on glass slides using Prolong Gold antifaded with DAPI and examined with multi-photon Leica SP5 microscope (UCSD School of Medicine Light Microscopy Facility). In few single reporter mice, we performed immunostaining and histochemical staining using the above methods as well. Tamoxifen treatment was also given in few wild type mice before staining with nNOS and ChAT antibodies to determine if it had any effect on the myenteric neurons. The ChAT and nNOS positive neurons were analyzed using maximum projection images, a volume rendering method for 3D data that projects in the visualized plane the voxels with maximum intensity that fall in the way of parallel rays traced from the viewpoint to plane of projection.

**Table 1:** Primary and secondary antibodies (made with 1% BSA/PBS) used for staining.

Primary antibody	Host species	Dilution	Source	Secondary antibody	Dilution	Source
nNOS	Rabbit	1:1000	Abcam AB5380	Goat anti rabbit 549	1:250	Abcam
ChAT	Goat	1:100	Abcam AB144P	Guinea pig anti goat 488	1:250	Millipore
PGP9.5	Mouse	1:500	Millipore AB72911	Goat anti mouse 647	1:250	Millipore
Hu	Mouse	1:100	Life Technologies A21271	Goat anti mouse 647	1:250	Millipore

### Whole-mount histochemistry (NADPH-diaphorase) staining

NADPH-diaphorase activity was detected histochemically, as described previously [13]. Briefly, the tissue was placed in 0.1 M phosphate buffer pH 7.4, containing 0.3% Triton X-100, 0.1 mg/ml nitroblue tetrazolium and 1.0 mg/ml /I-NADPH, at 37°C for 30–60 min. The reaction was halted by placing the sample in 0.1 M phosphate buffer. Stained samples were examined under a bright-field microscope. Histochemically stained samples were counterstained with DAPI 1:5000 to visualize nuclei. Samples were whole-mounted with either Fluoro-mount or 99% glycerol and covered with a cover slip.

Microscopic imaging was performed using a multi-photon, Leica SP5 confocal microscope. Z-stacks of myenteric plexuses were recorded using an upright laser scanning confocal microscope (SP5) with a water immersion 20x objective (0.9-μm-thick slices; z stack). Images were taken from the esophagus at 1, 2, 4, 6, 8, 10, 12, 14mm above the angle of HIS, intestine and colon. At least three images (high power field) were obtained from each animal at 20x magnification for cell counting. Tissue specimens were excited according to their specific excitation/emission characteristics. The detection pinhole was set for use with different objectives accordingly. Offset and gain settings were determined at the start of each experiment and kept constant throughout the image capturing time. The neurons were counted from these images manually using LAS AF software 4.3. Different magnification views were captured to visualize the neuronal process and cell interaction.

### Overall quantitative evaluation of neurons (ChAT/nNOS)

Neuron counting was performed manually on images taken under 20X magnification using Leica Application Suite Advanced Fluorescence Version 4.3. A 20x dry and/or water objective lens (numerical aperture 0.7 to 1) was used; the zoom factor was set at 1.72 in all scanning sessions. The neuron counts for only nNOS positive, only ChAT positive, both nNOS and ChAT positive and both nNOS and ChAT negative were obtained.

#### Statistical analysis

Data are shown as mean ± SEM. One-way ANOVA was used to compare differences between groups. p<0.05 was considered significant.

## Results

### Visualization of nNOS and ChAT positive neurons in the reporter mouse: Comparison with immunostaining and histochemical methods

**Fig 1:**
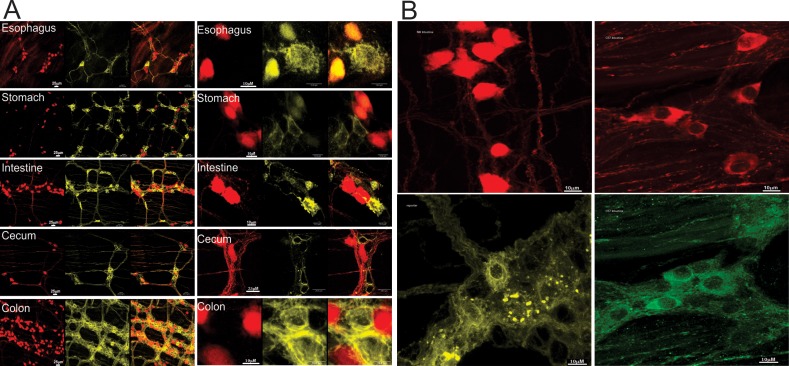
Representative Images. A: Images of nNOS and ChAT neurons from double reporter mice, B: Comparison between reporter (Left panel, top nNOS and bottom ChAT) and immunostain (Right panel: top nNOS and bottom ChAT) method. Note, that the nNOS is seen in both cytoplasm and nucleus in reporter mouse but only in the cytoplasm on immunostain. The ChAT positivity is seen only in the cytoplasm by both methods.

The Fig 1A shows representative images of nNOS and ChAT neurons in a double reporter mice from different regions of the GI tract at low (20x) and high (63x) magnification. Red colored cells in these images are nNOS containing neurons and green/yellow colored cells represent ChAT containing neurons. Both types of neurons and some but not all of their processes were visualized in the double reporter mouse in all examined areas of the GI tract, i.e., esophagus, stomach, ileum, cecum and colon. The red color of the nNOS neurons was seen in both, cytoplasm and nucleus in some cells, but only in the nucleus in other cells. On the other hand, the yellow/green color of ChAT neurons was seen only in the cytoplasm. The borders of nNOS neurons were distinct and fluorescence was strong. On the other hand, variability in the strength of yellow color fluorescence was observed in the ChAT positive neurons. Fig 1B shows comparison between findings from fluorescence reporter and immunostaining. The top row images compares nNOS positive neurons; on left from the reporter mice and on right from the C57 wild type mouse immunostained. Note that the nNOS positivity is seen in both cytoplasm as well as nucleus in the reporter mouse but only in the cytoplasm in the immunostained specimen. The bottom row compares cholinergic neurons by the two methods, the ChAT positivity is seen only in the cytoplasm by both methods. In the nNOS reporter mice, immunostaining for ChAT was performed and in the ChAT reporter mice immunostaining for nNOS was performed. In each of this mouse the percentage of nNOS and ChAT positive neurons were similar. More than 90% of the PGP 9.5 positive cells were positive for either nNOS or ChAT reporter.

**Fig 2:**
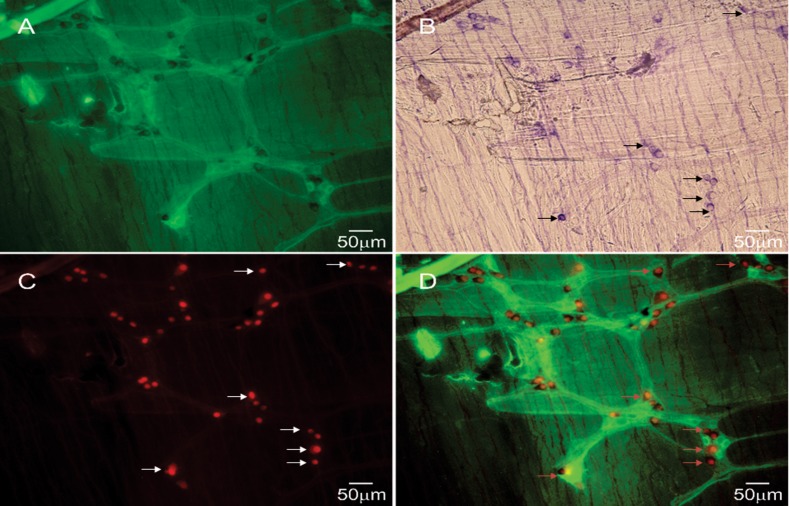
Double reporter mouse intestine treated with NADPH-d. Images excited using argon (488 nm, ChAT in A shown as green), HeNe1 (543 nm, nNOS in C shown as red), and bright light NADPH-d in B shown as purple). D merged all A, B, and C.

Fig 2 shows the small intestine of a double reporter mouse stained for NADPH-diaphorase activity to determine the agreement between the two methods of detection of nNOS neurons. In this image, ChAT positivity (A) is show in green, nNOS (C) in red, and NADPH diaphorase (B) in purple. The image in Fig 2D is merged from panels A, B, and C. The red fluorescence (nNOS) was seen in only those cells that also stained for NADPH diaphorase. The NADPH-diphorase staining was seen only in the cytoplasm but the fluorescent reporter was seen in both the cytoplasm and nucleus. The ChAT positive neurons were different from the NADPH-diaphorase positive neurons.

**Fig 3:**
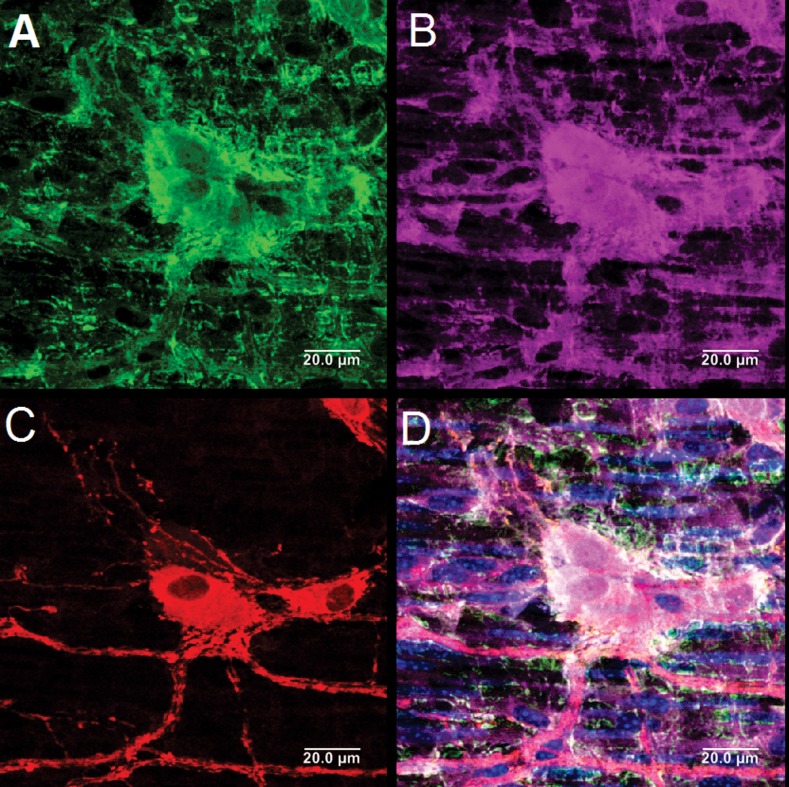
**C57 colon triple stained with ChAT (green, A), nNOS (red, C), and PGP 9.5 (purple, B). Nuclei stained with DAPI (blue).** D is the merge of all. Both ChAT and nNOS are also stained with PGP 9.5.

**Fig 4:**
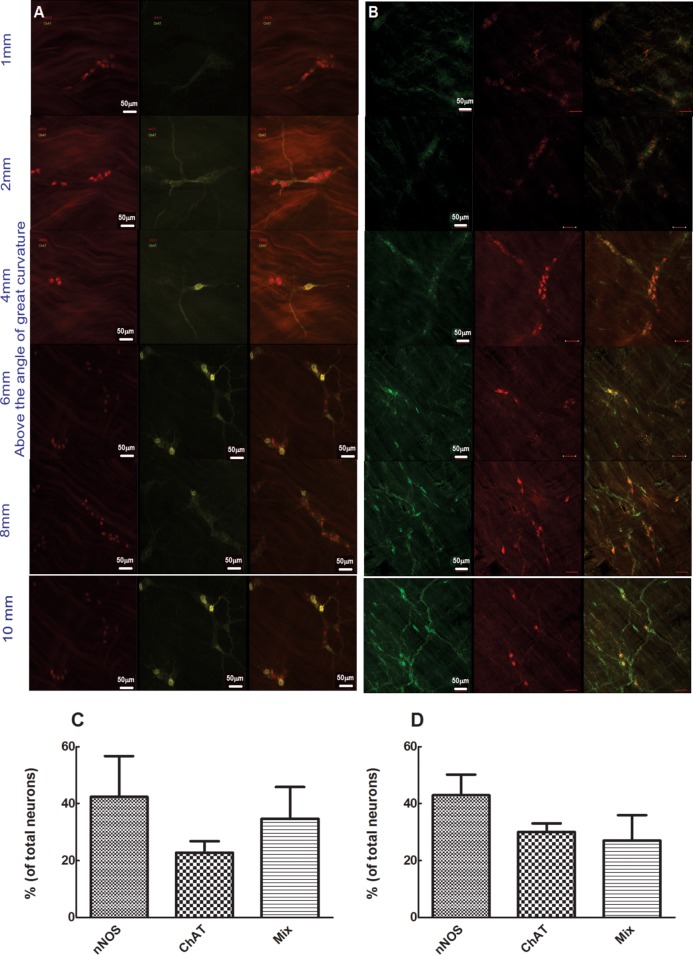
**Comparing double reporter mice (A) with C57 double stained with nNOS (red) and ChAT (green), (B) whole mount esophagus.** Images taken from 1mm, 2mm, 4mm, 6mm, 8mm, and 10mm above the angle of HIS. Note, red color cells represent nNOS and yellow color represent ChAT (in double reporter mice right side) and green color represents ChAT in immunostain. Different rows represent different locations along the length of esophagus. The images represent one of 7 mice. Scale bar = 50μm. Neuron counts summary (n = 7) shown in C for reporter mice and D for C57 mice.

Fig 3 shows a C57 wild type mouse colon triple stained with ChAT (green, A), nNOS (red, C), and PGP 9.5 (purple, B). Fig 4D is the merge of all. Nuclei were stained with DAPI (blue). Both ChAT and nNOS neurons are also stained with PGP 9.5. In general, negative control specimens revealed no staining in any of the samples. The nNOS and ChAT positive neurons were also positive for PGP 9.5 and Hu by immunostaining method. In the reporter mouse, it was easy to count nNOS positive neuron because of strong fluorescence and distinct outside borders. On the other hand, ChAT positivity even though present in the cytoplasm was not strong at times to distinctly define the cell margins. Therefore, neuronal counting is fairly accurate for nNOS neurons in the reporter mouse, but may be variable for ChAT neurons. Furthermore, the neuron counting was more accurate in the lower neuron density regions of the GI tract, i.e., esophagus. Overall neuronal density was > in colon > small intestine > esophagus.

### Regional differences in the ChAT positive and nNOS positive neurons along the gastrointestinal tract

**Fig 5:**
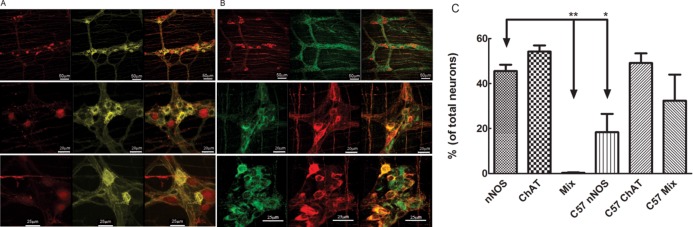
**Comparing double reporter mice (left panel, A) with C57 double stained with nNOS (red) and ChAT (green) of mice (right panel, B) whole mount intestine.** Note, red color cells represent nNOS and yellow represent ChAT (in double reporter mice left side) and green color represent ChAT in immunostain. C represents the summary data from 5–7 mice, shown as mean ± SEM.

Fig 4 shows the esophagus whole mount preparation of a double reporter mouse (A) compared with a C57 wild type mouse immunostained for nNOS (red) and ChAT (green) (B). Images were taken at 1, 2, 4, 6, 8 and 10 mm above the angle of HIS on the great curvature. The red color cells represent nNOS in the double reporter mice (right column) as well as immunostaining (left column), ChAT as yellow color in the reporter mice and green in the immunostaining method. Different rows represent different locations along the length of the esophagus. Neurons were observed as either single cells or small cluster of cells (ganglion). We observed that the density of nNOS neurons was similar throughout the length of the esophagus but more ChAT neurons were seen in the proximal esophagus compared to nNOS neurons. The fluorescent reporter for nNOS was located in the nucleus and for ChAT in the cytoplasm, while both markers were only found in the cytoplasm by immunostaining. By both methods, a small number of neurons were positive for both nNOS and ChAT. Neuron counts summary (n = 7) is shown in Fig 5C for the reporter mice and 5D for immunostain C57 wild type mice.

**Fig 6:**
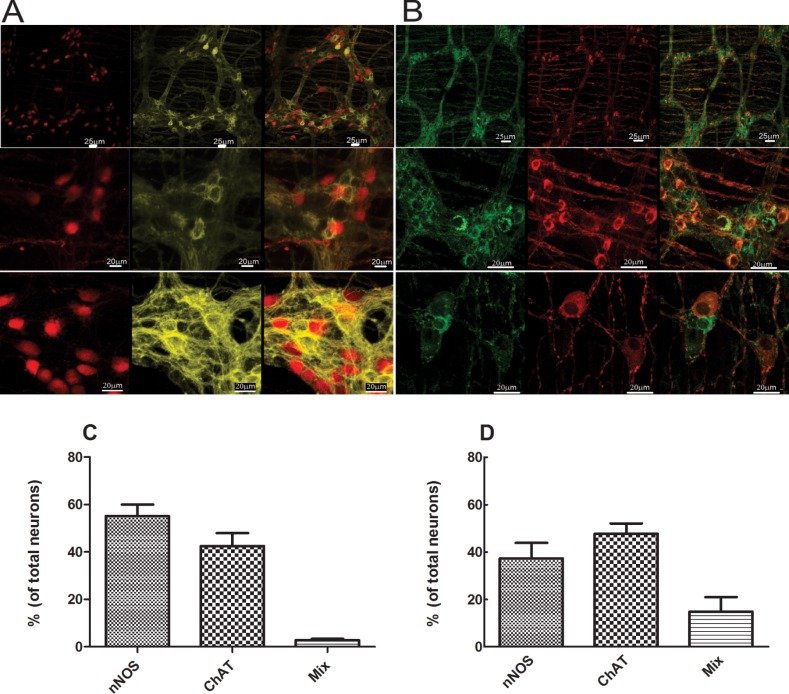
Comparing double reporter mice (top row) with C57 double stained with nNOS (red) and ChAT (green) of mice (bottom row) whole mount colon. Note red color cells represent nNOS and yellow color represents ChAT (in double reporter mice left side) and green color represent ChAT in immunostain. C (double reporter mice) and D (C57 immunostain) represent the summary data from 5–7 mice, shown as mean ± SEM.

Fig 5 shows a comparison between double reporter mice (column A) and immunostain method for nNOS (red, column B) and ChAT (green, column B) in whole mounts of the small intestine. The red color represents nNOS in both the reporter and immunostain method, ChAT-positive cells are yellow in the double reporter mice and green in the immunostain. The exclusive presence of ChAT in the cytoplasm of neurons is similar between the two methods. On the other hand, the appearance of nNOS neurons differed in the two detection methods, with nNOS fluorescence present in the cytoplasm and nucleus in reporter mice, but only in the cytoplasm on immunostain. Fig 6C displays summary data from 5–7 mice (mean ± SEM). nNOS single-positive neurons were detected in lower number by immunostain compared to fluorescent reporter analysis, while nNOS/ChAT double-positive neurons were observed in high numbers by immunostain method compared to the fluorescent reporter mice. These difference are significant (p<0.05; one-way ANOVA).

**Fig 7:**
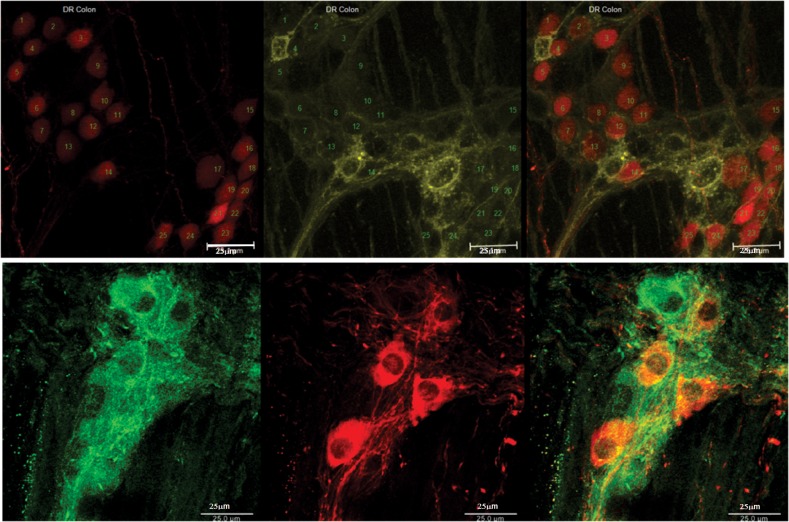
Images of co-localized nNOS and ChAT, double reporter (top row) and immunofluorescence (bottom row). Cells marked with numbers 12 and 24 in the top row are examples of co-localized nNOS and ChAT in the same neuron.

Fig 6 shows whole mounts of the colon, comparing double reporter mice (column A) with the immunostain method in the C57 wild type mice stained for nNOS (red) and ChAT (green) (column B). Note, the red color cells represent nNOS and yellow color represents ChAT in the double reporter mice (left side) and green in the immunostain (right side). The ChAT neurons appear similar between the two methods, ChAT positivity is seen only in the cytoplasm and not the nucleus. On the other hand, the nNOS positivity in the reporter mice is positive in the cytoplasm and nucleus. Fig 7C (double reporter mice) and 7 D (C57 immunostaining) represent summary data from 5–7 mice. There is no difference (p>0.05, One-way ANOVA) between the nNOS, ChAT, and double-positive neurons by two methods. Fig 7 shows examples of the nNOS and ChAT neurons by the two methods (top row for double reporter and bottom row for immunostain method in the wild type mouse).

**Fig 8:**
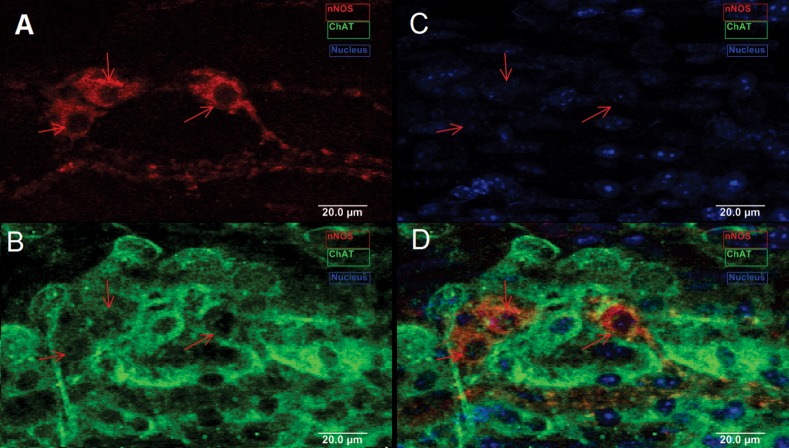
Images show example of nNOS and ChAT neurons in the wild type mice with treatment with tamoxifen of the colon. Note there isno effect on the appearance of two types of neurons. A: nNOS, red; B: ChAT, green; C: Nucleus, blue; D: A merge from A to C images.

We also determined if tamoxifen treatment had any effect on the myenteric neurons since to visualize nNOS neurons in the reporter mouse tamoxifen was administered. Wild type mice were treated with tamoxifen in a manner similar to the reporter mouse. Fig 8 shows examples of ChAT and nNOS neurons in the untreated and treated mouse and we found no difference.

## Discussion

This paper reports on the construction and validation of a powerful new genetic tool for the studies of the enteric nervous system. We generated dual fluorescent reporter mice for simultaneous detection of nNOS and ChAT positive neurons in the gastrointestinal tract. Both types of neurons were well visualized in the reporter mice and the results were generally comparable to the findings obtained by standard immunostaining method, which is considered to be the current “gold standard”. The visual appearance as well as the number of cells is not different by the two techniques. Even the size of cells and their processes appear to be similar when studied by the two techniques. We found that a small percentage of neurons are positive for both nNOS and ChAT proteins when visualized by the two methods of detection that we studied.

Udit et al, in year 2013[12] summarized all the reporter mice described in the literature for various proteins expressed in the enteric nervous system. Mercer and colleagues were the first to report B-gal reporter mouse that labels non-adrenergic neurons of the sympathetic nervous system in 1991[14]. The eGFP labelled ChAT reporter mouse was first described by Gong et al. in 2003[15]. More recently, Hao et al [11] and Erickson et al [16] studied development of myenteric cholinergic neurons in the ChAT-Cre, YFP reporter in the gut of embryonic and post-natal mice [11]. They observed that there are differences in the expression of ChAT by immunostaining and the genetic reporter technique. The ChAT reporter expression of cholinergic neurons was seen at an earlier stage of development than the pan neuronal marker. Ours is the first report of nNOS reporter mouse and the double reporter mouse that expresses both ChAT and nNOS proteins in the enteric neurons. The major advantage of having such an experimental model is that it will make the study of the effects of various pathologies on the enteric nervous system significantly easier because approximately 95% of the enteric neurons contain ChAT and or NOS. Immunostaining techniques are cumbersome and they do not always produce reproducible results; the results are dependent on the quality of antibody. Furthermore, the antibodies may not diffuse through the whole thickness of the tissue and thus may not give reliable results.

We observed that throughout the GI tract the nNOS positivity in neurons was present only in the cytoplasm when visualized by immunostain method but it was seen in the cytoplasm as well as the nucleus in the reporter mouse. The nuclear staining in the reporter mouse actually appeared more intense than in the cytoplasm and in some neurons nNOS was only present in the nucleus which gave the false appearance that these cells were smaller in size than the ChAT neurons. The reason for differences between the two methods is most likely related to the properties of nNOS and fluorescent marker Td Tomato. Expression of both proteins is controlled by nNOS promoter and therefore same cells should be positive for both proteins. The nNOS is a cytoplasmic protein and should stay only in the cytoplasm. If the Td Tomato were a fusion protein with nNOS one would expect Td Tomato—nNOS staining would behave exactly like nNOS and be only present in the cytoplasm. In reality though Td Tomato is not a fusion protein and thus does not show up exactly like nNOS and may diffuse freely across the cell and even into the nucleus. The degree of Td Tomato diffusion most likely depends on the type and speed of tissue fixation. Given that the Td Tomato and nNOS are expressed in the same cells because they share the common nNOS promoter, overall interpretation with regards to identification and number of neurons is not affected by the location of fluorescence. However it does make the details a bit unexpected. Looking at the figures of Hao et al[11] for ChAT reporter mouse using BAC methodology that we used for the nNOS reporter protein, ChAT positivity can be seen in both the cytoplasm as well as the nucleus, a result consistent with our findings.

We carefully assessed different regions of the GI tract for the presence of neurons that contained both ChAT and nNOS and found that small number of neurons in all regions of GI tract were positive for both, in the reporter mouse method as well as the immunostain method. The neurons positive for both nNOS and ChAT have been described in all species studied, including mice, guinea pigs and humans by several investigators[9, 17]. On close inspection of these cells using confocal microscopy, we could not rule out the possibility that the co-localization of nNOS and ChAT may be a visual artifact related to close approximation of the two different cells giving an appearance of one cell. We found some differences in count of neurons in different regions using the two techniques, e.g., in the small intestine the number of ChAT and nNOS positive neurons is greater by the immunostain method compared to the reporter mouse. The reason for the above is not clear; however, the neuron counts for the nNOS and ChAT was not different in the esophagus and colon.

In summary, we describe a genetically engineered mouse that expresses nNOS and ChAT and illuminates 95% of the neurons of the enteric nervous system. The data provided in this paper are mostly descriptive, which may be considered as one of the limitation of our study. However, we propose that this double reporter mouse for nNOS and ChAT is a powerful tool in the study of the effects of specific diseases, e.g., diabetes mellitus, alterations in the gut microbiome and intestinal obstruction on the enteric nervous system, without having to rely on the antibodies and immunostaining methodology.
